# Soil Disturbance Affects Plant Productivity via Soil Microbial Community Shifts

**DOI:** 10.3389/fmicb.2021.619711

**Published:** 2021-02-01

**Authors:** Taylor J. Seitz, Ursel M. E. Schütte, Devin M. Drown

**Affiliations:** ^1^Department of Biology and Wildlife, University of Alaska Fairbanks, Fairbanks, AK, United States; ^2^Institute of Arctic Biology, University of Alaska Fairbanks, Fairbanks, AK, United States

**Keywords:** permafrost thaw, microbial communities, boreal forest, metagenomics, plant growth

## Abstract

Recent advances in climate research have discovered that permafrost is particularly vulnerable to the changes occurring in the atmosphere and climate, especially in Alaska where 85% of the land is underlain by mostly discontinuous permafrost. As permafrost thaws, research has shown that natural and anthropogenic soil disturbance causes microbial communities to undergo shifts in membership composition and biomass, as well as in functional diversity. Boreal forests are home to many plants that are integral to the subsistence diets of many Alaska Native communities. Yet, it is unclear how the observed shifts in soil microbes can affect above ground plant communities that are relied on as a major source of food. In this study, we tested the hypothesis that microbial communities associated with permafrost thaw affect plant productivity by growing five plant species found in Boreal forests and Tundra ecosystems, including low-bush cranberry and bog blueberry, with microbial communities from the active layer soils of a permafrost thaw gradient. We found that plant productivity was significantly affected by the microbial soil inoculants. Plants inoculated with communities from above thawing permafrost showed decreased productivity compared to plants inoculated with microbes from undisturbed soils. We used metagenomic sequencing to determine that microbial communities from disturbed soils above thawing permafrost differ in taxonomy from microbial communities in undisturbed soils above intact permafrost. The combination of these results indicates that a decrease in plant productivity can be linked to soil disturbance driven changes in microbial community membership and abundance. These data contribute to an understanding of how microbial communities can be affected by soil disturbance and climate change, and how those community shifts can further influence plant productivity in Boreal forests and more broadly, ecosystem health.

## Introduction

With nearly 85% of the land in Alaska underlain with discontinuous permafrost, Alaska is particularly vulnerable to large-scale ecosystem changes due to climate change-driven permafrost thaw and resulting soil disturbance (Department of Natural Resources: Alaska Division of Geological and Geophysical Surveys, 2020). As foundational abiotic factors such as nutrient availability and soil hydrology begin to change, they in turn affect ecosystem processes including succession and productivity, that regulate plant and microbial communities present in the disturbed soils (Schuur and Mack, 2018). Recent research has turned to exploring the effects of climate change on microbial communities residing in soil and permafrost (Jansson and Hofmockel, 2018). As permafrost thaws and soil disturbance events occur, microbial communities undergo shifts in membership composition and biomass, as well as in functional diversity (Mackelprang et al., 2011; Coolen and Orsi, 2015; Schuur et al., 2015; Schütte et al., 2019). As these smaller scale changes develop, it is important that we build a better understanding of how microbial communities in northern latitude soils affect larger scale processes such as plant growth.

Permafrost is frozen ground (rock, ice, soil) that remains at less than 0°C for more than 2 years. Permafrost underlies approximately 26% of terrestrial ecosystems globally and is a critical structural element that holds large potential for altering ecosystem composition through thawing and melting of ice wedges (Schuur and Mack, 2018). It has been estimated that the carbon stored in permafrost accounts for more than 2.5 times as much as the atmospheric carbon pool (Schuur et al., 2007, 2015). As permafrost thaws, previously frozen organic matter, nutrients, water, and microbes are exposed and reintroduced into actively cycling pools of carbon and nutrients (Chapin et al., 2006). Plant roots interact within the active layer of soil which sits above the permafrost and undergoes seasonal cycles of freezing and thawing (Christensen et al., 2004; Schuur et al., 2015). The depth of the active layer defines what nutrients, rooting space, and water are available to plants which can in turn affect community succession and composition.

Previous research has found evidence that the composition, diversity, and biomass of microbial communities can become altered in locations that are undergoing permafrost thaw and disturbance events such as thermokarst formation (Schuur et al., 2007; Mackelprang et al., 2011; Monteux et al., 2019). The atmospheric release of carbon trapped in soils following permafrost thaw is mediated by soil bacterial and fungal respiration. These organisms have been surviving in the active layer and frozen soil below for millennia, either frozen or respiring at extremely low rates, using the cold organic matter as an energy source (Tripathi et al., 2018). As the permafrost thaws, soil microbes exit dormancy and microbial communities gain access to large pools of previously frozen carbon and nutrients (Burkert et al., 2019). As the activity of the microbial communities increases with a warming climate, microbial effects may be more complicated than merely releasing greenhouse gases into the environment through respiration. These microbes can also cause alterations and feedback to ecosystem nutrient cycling and may in turn drive alterations to the plant community in associated soils (Natali et al., 2012; Du Toit, 2018).

Plant-microbe interactions within the rhizosphere are known determinants of plant health and productivity, and consequently, plant growth (Reynolds et al., 2003; Bever et al., 2010; Mangan et al., 2010). Interactions between microbes and plants are critical for the acquisition and cycling of nutrients such as biological nitrogen fixation and phosphate solubilization (de Souza et al., 2015). Highly studied environments such as agricultural production have shown evidence that small changes to soil microbiomes largely determine the success of plant growth and development (Van Wees et al., 2008; Lugtenberg and Kamilova, 2009). Wei et al. (2019) showed that within an agricultural monoculture system, the composition of the initial soil microbial inoculant predetermined whether a crop succumbed to disease or survived. Previous studies focused in the arctic and sub-arctic have demonstrated that soil microbes are important drivers of plant succession, relative abundance, and productivity (Schuur et al., 2007; Natali et al., 2012). As physical and chemical properties of soil such as temperature and pH are changing, the composition of microbial communities present in the active layer changes as well. Permafrost thaw could induce now active microbes to transform newly available organic matter into gaseous products such as CO_2_ and CH_4_, and metabolites that are now accessible to plants and other organisms living in and interacting with active layer soils (Graham et al., 2012). With the availability of newly released nutrients and the production of metabolites, soil microbes then have the potential to shape above ground plant communities by mediating and partitioning soil resources (Ho et al., 2017). Not only do beneficial microbes assist in nutrient accessibility, but they can also improve plant performance in a variety of systems by conferring resistance to pathogens.

Here we investigate how active layer soil microbial communities affect above ground plant communities, indicating that microbes play a role in climate change driven alterations to Alaskan plant communities. We hypothesized that plants inoculated with microbial communities from disturbed soils associated with greater permafrost thaw will experience lower levels of plant productivity compared to plants inoculated with microbes from less disturbed soils. We tested this hypothesis by conducting a plant growth experiment on boreal plant species inoculated with microbes from soils with different degrees of permafrost thaw and assessed soil microbial community composition.

## Materials and Methods

### Sample Site Description and Soil Collection

The Fairbanks Permafrost Experiment Station (FPES) is located in interior Alaska (64.877°N, 147.670°W) and is part of the US Army Corps of Engineers Cold Regions Research and Engineering Laboratory (CRREL). FPES is divided into three Linell plots (Linell, 1973; Douglas et al., 2008), each 3,721 m^2^: undisturbed (UD), semi disturbed (SD), and most disturbed (MD) (Supplementary Figure 1). When these three plots were created in 1946, the first plot, UD, was left untouched. The second plot (SD) was cleared of trees and above ground vegetation by hand, but the roots and other organic material were left intact. The third plot (MD) was stripped of all vegetation and surface level organic material down to mineral soil. Over the next 25 years, total stripping (MD) led to permafrost thaw and degradation to 6.7 m below surface level, and partial stripping (SD) led to thaw 4.7 m below surface level. These plots were created to simulate soil disturbance events and to then identify potential ecological effects.

Each plot is representative of the subarctic taiga forest. The undisturbed plot is covered by a dense black spruce stand (*Picea mariana*) with intermittent white spruce (*Picea glauca*). Its understory is dominated by Labrador tea (*Rhododendron groenlandicum*) and low-bush cranberry (*Vaccinium vitis-idaea*) with a ground cover of continuous feather and *Sphagnum* moss and lichen. The surface soils are moderately moist and can be classified as mesic, with an average soil organic layer thickness of 35 cm and a consistent thaw depth of 85 cm (Douglas et al., 2008; Johnstone et al., 2008). The semi-disturbed plot is covered by a mixed stand of Alaskan birch (*Betula neoalaskana*), willow (*Salix* sp.), black spruce, and white spruce, with a developing understory of moss and shrubs. The trees at the semi-disturbed plot are taller than those in the most disturbed. The most disturbed plot is covered by a mixed stand of Alaskan birch, willow, and young black spruce. The understory is a mixture of moss and grass (Douglas et al., 2008).

We collected 48 soil cores from FPES on 28 May 2018, 16 individual cores at each FPES treatment level. Using an established grid layout of FPES, we took cores from four selected quadrats per plot to demonstrate the fine-scale heterogeneity of the sample site. At each quadrat we took four samples at the corners, approximately 1 m apart. We utilized a sterile technique to sample soil and at each sampling point, the top layer of moss and vegetation was removed. We used a soil corer (4.5 cm diameter by 10 cm height) to collect the top 10 cm of soil, which were stored in a cooler throughout the duration of sample collection. We then transported the cores back to the lab where each was homogenized and stored at 4°C to be used 10 days later for soil inoculants and between 7 and 45 days later for DNA extractions.

### Greenhouse Experimental Design and Sampling

To test how the sampled microbial communities affect plant growth, we grew all plants in a climate-controlled greenhouse, within autoclaved, nutrient-poor soils containing soil microbial inoculum. The soil microbial inoculants were obtained from FPES for the three treatment levels: UD, SD, and MD. We also included a sterile inoculant (ST), an autoclaved mixture of UD, SD, and MD soils.

**FIGURE 1:**
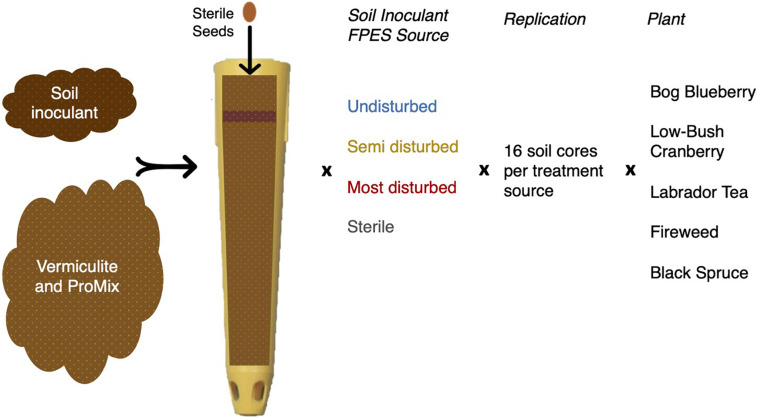
Overview of experimental design for the greenhouse plant growth experiment. Soil microbial inoculant was sourced from FPES soil cores and potting soil is a sterile mixture of vermiculite and ProMix.

We conducted the growth experiment in the Institute of Arctic Biology Greenhouse at the University of Alaska Fairbanks (UAF), consisting of five plant species: *Vaccinium vitis-idaea* (low-bush cranberry), *Vaccinium uliginosum* (bog blueberry); *Picea mariana* (black spruce); *Ledum groenlandicum* (Labrador tea); and *Chamerion angustifolium* (fireweed), hereafter all plants will be referred to by their colloquial names. We obtained all seeds from Alaskan sources, and we surface-sterilized them prior to planting. On 11 June 2018, we placed 5–10 seeds of one plant type into a pot (SC10 Cone-tainers, Ray Leach; United States) filled with a mixture of densely packed sterile vermiculite and Canadian peat (ProMix), with 1.5 g of soil containing the treatment microbial soil inoculant (Figure 1). Sixty-four pots were planted for each plant type, with 16 unique soil inoculants per each of four treatments, for a total of 320 pots. We randomized all pots across seven RL98 trays (Ray Leach, United States) to reduce possible bias from temperature or light gradients within the greenhouse. Plants were maintained under 12 h light cycles and watered once daily.

Following the first month of growth past germination, we measured the plants biweekly for the first 3 months, recording their leaf count and height to the nearest millimeter. We stopped black spruce needle counts in November 2018, when needle counts exceeded 200 and we were unable to accurately measure. By October 2018, all the fireweed (*n* = 61) had started to decline in height measurements, so on 10 October 2018, we clipped above ground living plant material at the soil surface and then oven dried plants 55°C to a constant mass before determining biomass produced. The bog blueberry, black spruce, Labrador tea, and low-bush cranberry continued to grow and be measured monthly. We then harvested above ground biomass per plant type in March 2020, and below ground biomass in April 2020. We oven dried plants at 55°C to a constant mass before weighing.

### Statistical Analysis

All statistical analyses were performed in R version 3.5.2 (R Core Team, 2020). In order to test the effects of the microbial inoculant treatments during the plant growth experiment, we used the Shapiro-Wilk test and Levene’s test to check ANOVA assumptions. We log-transformed growth measurements (leaf count, height, biomass) per plant type if it improved normality and reduced heteroscedasticity. We used a one-way ANOVA (Car R package, v3.0.3) to evaluate the significance of soil inoculant on each plant growth measurement within each plant type. We used a Tukey HSD (Agricolae R package, Mendiburu and Yaseen, 2009, v1.3.2) *post hoc* test to identify significant effects of specific inoculant treatments on growth within each plant type. We visualized differences using the R package ggplot2 v3.2.1).

### DNA Extractions and Metagenomic Sequencing

In order to analyze the microbial communities used to inoculate plants in the growth experiment, we performed shotgun metagenomic sequencing on each individual core. To do this, we extracted and purified total genomic DNA from approximately 250 mg of soil per homogenized soil core using the DNeasy PowerSoil kit (Qiagen; Germany) following manufacturer instructions. We quantified the yield and the quality of the DNA extracted using a NanoDrop One spectrophotometer (Thermo Fisher Scientific; United States) and a Qubit (Thermo Fisher Scientific; United States). Following DNA extractions, we randomly divided the 48 cores into four sequencing runs. We prepared the DNA sequencing library using the Oxford Nanopore Technologies Ligation Kit with Native barcodes to multiplex 12 samples (SQK-LSK109, ONT; United Kingdom). We diluted sample DNA to 400 ng for input and followed the kit according to the manufacturer instructions. We then sequenced DNA using a MinION and R9.4.1 and R9.5 flow-cells (FLO-MIN106) (Supplementary Table S1). The sequencing runs each lasted 48 h.

### Metagenomic Analyses

We base called the raw data using Guppy v3.6.1 (ONT) specifying the high accuracy model (Supplementary Table S1). We then demultiplexed samples with the Guppy barcoder using parameters to discard sequences with middle adapters and to trim barcodes. We used Filtlong v0.2.0^1^ to control for length (≥50 bp) and quality (Q) score (≥10). Following quality control, we detected taxa with a *k*-mer based approach using Kraken 2 (v.2.0.9-beta; Wood et al., 2019) and subsequently estimated abundance using Bracken (v.2.6.0; Lu et al., 2017). We used a reference database prepared with archaeal, bacterial, fungal, protozoal, and viral sequences from RefSeq on 22 May 2020 (Nicholls et al., 2019). We merged sample Bracken reports and then generated heat maps based on the most prevalent bacterial phyla, families, and genera with gplots v.3.0.3 in R. We excluded reads that were classified as fungal for any further analyses, due to the lack of breadth in our database.

In order to identify biomarkers within the microbial communities we used the linear discriminant analysis (LDA) effect size (LEfSe) method (Segata et al., 2011). This method compares across all taxonomic levels and identifies differentially abundant features between biological classes using a non-parametric factorial Kruskal-Wallis sum-rank test. LEfSe then uses LDA to estimate the effect sizes of each feature. To accomplish this, we uploaded our Bracken results to the Galaxy web platform^2^ (Afgan et al., 2018) where we then used the Huttenhower Lab workflow^3^ for LEfSe analysis. We used an LDA threshold of 4.0 and significance α of 0.01 to detect biomarkers. Following the LEfSe analysis, we estimated Pearson’s correlation values (stats R package v3.6.1), r, between plant productivity measures and the significant biomarkers that LEfSe identified.

## Results

### Plant Growth Experiment

**FIGURE 2:**
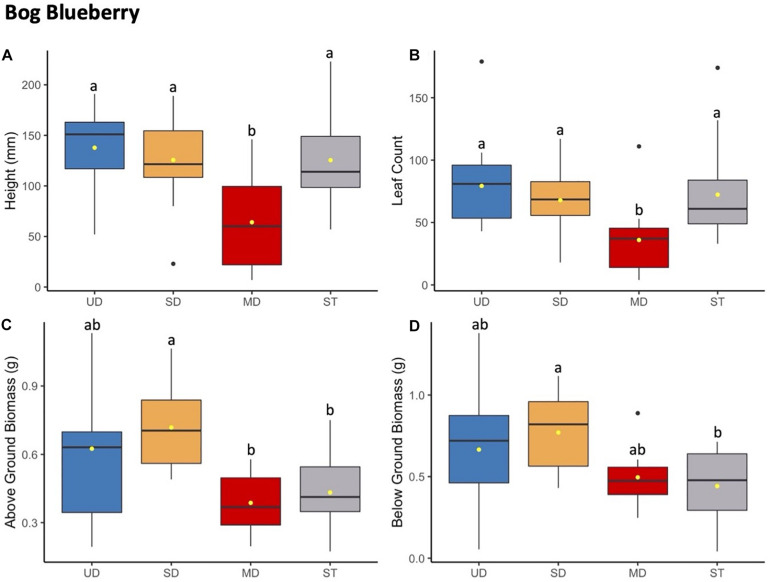
Bog blueberry plant height (mm) **(A)** and leaf count **(B)** at 184 days since planting. Above ground **(C)** and below ground **(D)** biomass after 1.5 years since planting. Boxplots represent median, and the upper and lower quartiles. Yellow circles represent mean value for each group. Tukey HSD *post hoc* comparisons are denoted with lowercase letters above boxplots.

**FIGURE 3:**
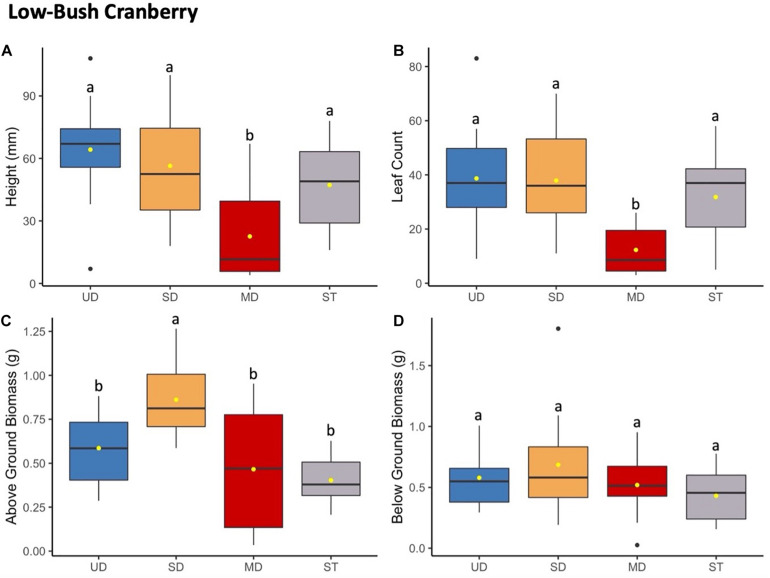
Low-bush cranberry plant height (mm) **(A)** and leaf count **(B)** at 184 days since planting. Above ground **(C)** and below ground **(D)** biomass after 1.5 years since planting. Boxplots represent median, and the upper and lower quartiles. Yellow circles represent mean value for each group. Tukey HSD *post hoc* comparisons are denoted with lowercase letters above boxplots.

**FIGURE 4:**
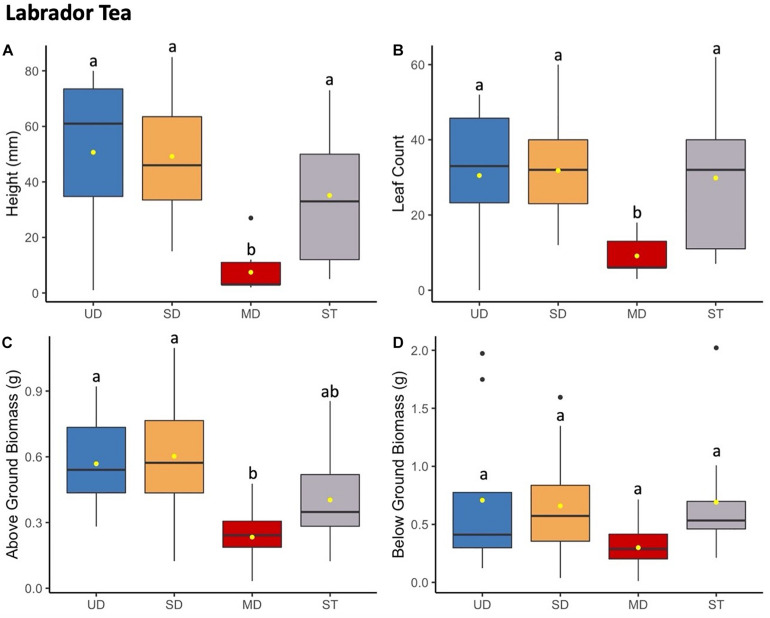
Labrador tea plant height (mm) **(A)** and leaf count **(B)** at 184 days since planting. Above ground **(C)** and below ground **(D)** biomass after 1.5 years since planting. Boxplots represent median, and the upper and lower quartiles. Yellow circles represent mean value for each group. Tukey HSD *post hoc* comparisons are denoted with lowercase letters above boxplots.

**FIGURE 5:**
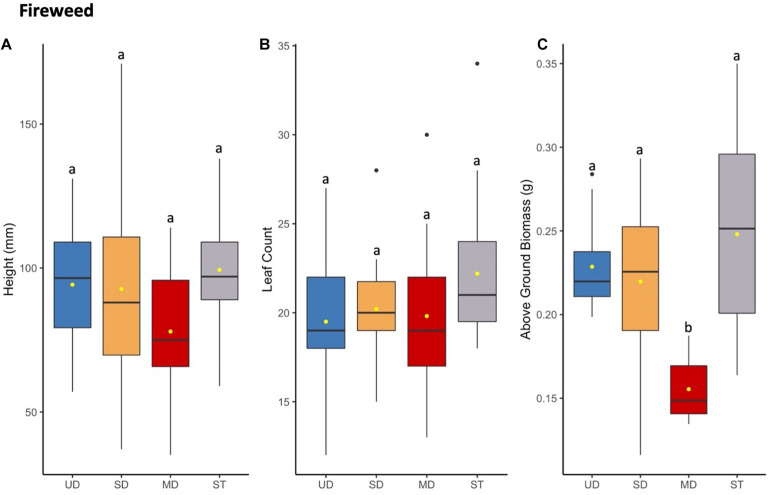
Fireweed plant height (mm) **(A)** and leaf count **(B)** at 121 days since planting. Above ground **(C)** biomass of plants after 165 days since planting. Boxplots represent median, and the upper and lower quartiles. Yellow circles represent mean value for each group. Tukey HSD *post hoc* comparisons are denoted with lowercase letters above boxplots.

**FIGURE 6:**
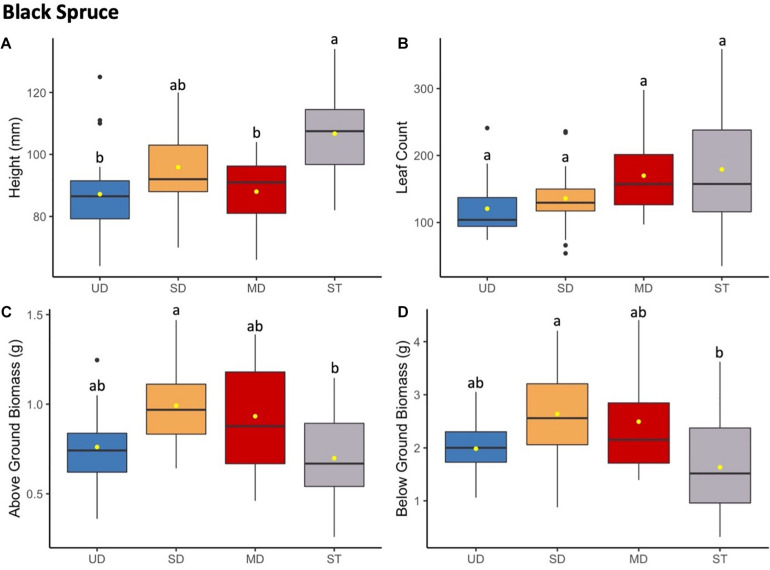
Black spruce height (mm) **(A)** and leaf count **(B)** at 184 and 121 days since planting, respectively. Above ground **(C)** and below ground **(D)** biomass after 1.5 years since planting. Boxplots represent median, and the upper and lower quartiles. Yellow circles represent mean value for each group. Tukey HSD *post hoc* comparisons are denoted with lowercase letters above boxplots.

Our results indicate that soil microbial communities from across the thaw gradient differentially affected plant productivity (Figures 2–6 and Supplementary Tables S3–S8). Most plant types responded negatively when grown in soils inoculated with microbial communities from the most disturbed FPES soil compared to either inoculants from semi disturbed or undisturbed soils, or the sterile treatment (Supplementary Figure S2 and Supplementary Table S8).

#### Bog Blueberry

Bog blueberry had reduced growth in height and leaf count when grown in soils inoculated with microbial communities from the MD treatment site compared to plants growth with inoculants from UD, SD, or ST soil (Figure 2). This trend was consistent across height and leaf count starting at the third through the final measurement point (Supplementary Figure S2). While the mean height and leaf count of bog blueberry grown in MD treatment was significantly smaller compared to bog blueberry grown under the SD, UD, or ST treatments (Supplementary Table S3), there was no significant difference between height or leaf number for bog blueberry plants grown in UD, SD, or ST treatments. While the source of inoculant was a significant factor in above ground and below ground biomass, the responses did not follow the same trends as height and leaf count. For above ground biomass the *post hoc* Tukey HSD test showed that SD plants had more mass on average and differed significantly from MD and ST, SD plants did not differ significantly in biomass production (Supplementary Table S8).

#### Low-Bush Cranberry

Low-bush cranberry showed the same trends to bog blueberry when grown in soils from the most disturbed treatment site (Figure 3). Low-bush cranberry height and leaf count significantly decreased when grown with inoculant from the MD site compared to when grown with inoculant from the SD or UD sites, or with ST inoculant (Supplementary Tables S4, S8). There was no significant difference in either height or leaf count between low-bush cranberry grown in UD, SD, or ST treatments. Above ground biomass increased when plants were grown with soil from the SD compared to plants inoculated with UD, MD, and ST treatments. Low-bush cranberry below ground biomass did not differ significantly depending on the soil inoculant treatment.

#### Labrador Tea

Labrador tea plants displayed nearly identical trends to both bog blueberry and low-bush cranberry when grown in soils inoculated with microbial communities from the most disturbed treatment site (Figure 4). At the time of final measurements, the mean height and leaf count of Labrador tea grown in the MD treatment was significantly smaller compared to low-bush cranberry grown SD, UD, or ST treatments (Supplementary Table S5). There was no observed difference between Labrador tea grown in UD, SD, or ST treatments. Labrador tea above ground biomass showed that MD plants weighed in at a significantly smaller amount than plants grown in soils inoculated with UD or SD treatments. No differences were observed between below ground biomass measures (Supplementary Table S8).

#### Fireweed

In contrast to cranberry, bog blueberry, and Labrador tea, fireweed did not show a significant difference in mean leaf count or height (Figure 5); however, fireweed plants grown in inoculant from MD site showed a significant decrease in above ground biomass compared to those grown in UD, SD, or ST treatment soils (Figure 5 and Supplementary Tables S6, S8).

#### Black Spruce

Black spruce showed no consistent response patterns to microbial inoculant for growth and biomass measures (Figure 6 and Supplementary Table S7). Black spruce plants grew significantly taller when grown with the sterile inoculant (ST) compared to UD, SD, or MD inoculants. Below ground and above ground biomass showed both showed a similar pattern of ST plants measuring at a significantly lower biomass compared to SD plants, and with MD and UD plants showing no significant differences in mass. For leaf (needle) count, no significant differences were observed across all treatment inoculant groups. Leaf count was discontinued and excluded from analysis after needle numbers exceeded 200 for all plants and was not able to be accurately measured.

### Microbial Community Analysis

We sequenced 48 metagenomes through four multiplexed MinION runs, and after demultiplexing and quality control, total sample reads showed a mean read length of 2,594 bp, a N50 of 5,531 bp, and a mean quality score of 13 (Supplementary Table S2). All samples had an average yield of 373 Mbase (range: 182–737 Mbase) with an average read count of 143 K reads (range: 53–299 K). The analysis using Kraken 2 and then Bracken resulted in a mean of 41% (range: 30–60%) of reads being classified as bacterial (60,034 reads). The percent classified did not show a correlation to the read depth of the sample (Supplementary Figure S3).

**FIGURE 7:**
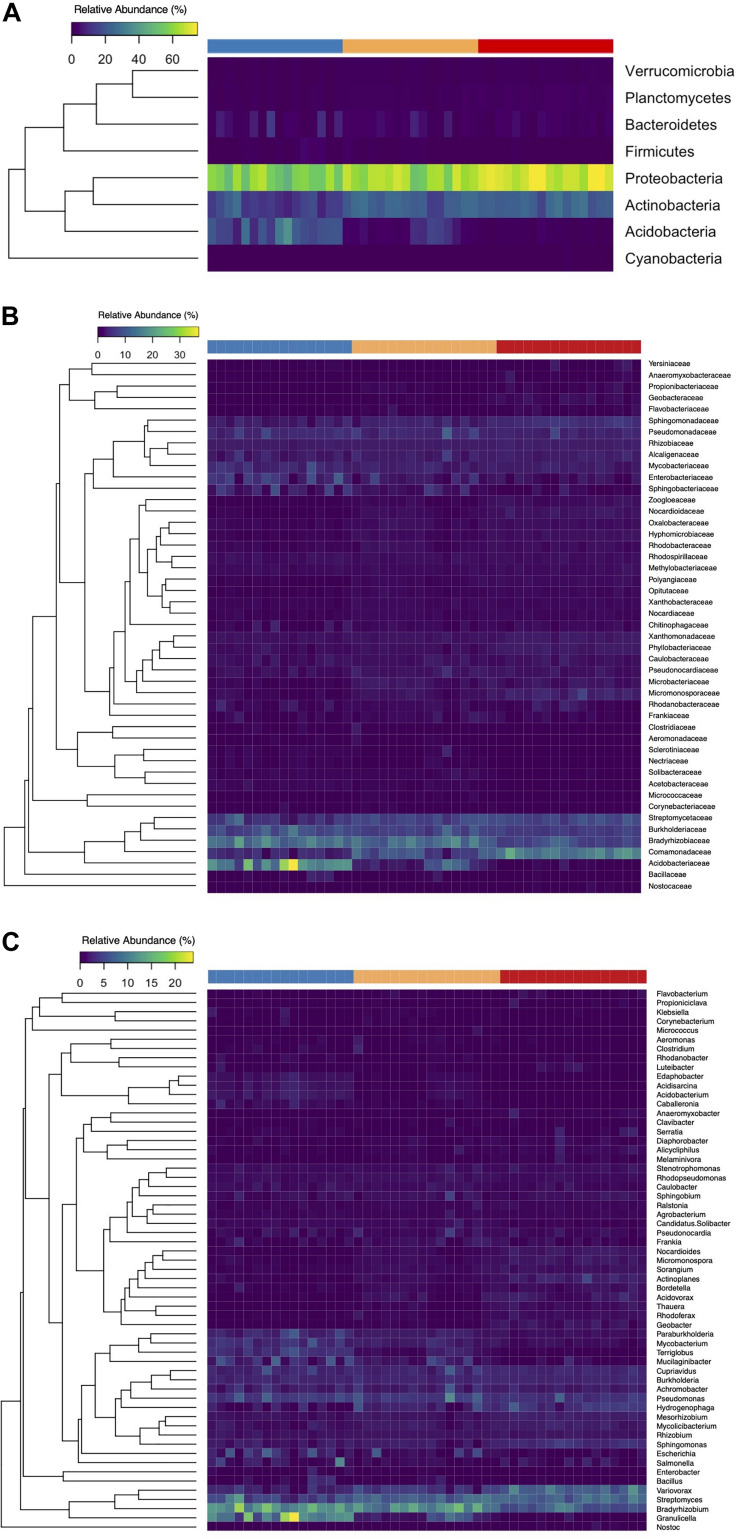
Heat maps of the relative abundances of bacterial phyla **(A)**, families **(B)**, and genera **(C)** identified at relative abundances >1% per soil core (columns). The top row signifies the level of (FPES) soil disturbance of each core: UD, blue; SD, yellow; MD, red.

Following classification via Kraken 2 and Bracken, we identified 24 bacterial phyla within our microbial communities. A majority of samples across the three levels of disturbance were largely dominated by Proteobacteria and Actinobacteria with mean relative abundances of 62.9 and 19.9%, respectively. Of the 24 bacterial phyla identified, eight were present at relative abundances higher than 1% (Figure 7A). We observed a general shift in community membership and abundance across the disturbance gradient, with both the UD and MD soil cores showing variation in the dominant taxonomic groups present, all the way down to the genus level (Figures 7B,C). Among the 59 genera present at more than 1% relative abundance, *Bradyrhizobium* and *Streptomyces* were present at the highest relative abundances, with means of 10.2 and 6.9%, respectively.

To further analyze the taxonomic shifts, we utilized the LEfSe method to identify any biomarkers across samples. Out of only the bacterial reads, we identified 13 differentially abundant biomarkers across the phylum to family level within our dataset (Table 1). Four of the biomarkers, that all fall under the same taxonomic branch of the phylum Acidobacteria and down to the family *Acidobacteriaceae* were found to be overrepresented in UD soil communities. Nine biomarkers were found to be overrepresented in MD soil communities, including members of the phyla Proteobacteria and Actinobacteria (Table 1).

**TABLE 1:** Pearson’s correlation estimates (*r*) between plant productivity and microbial community biomarkers.

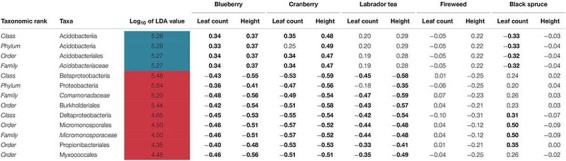

*Pearson correlation estimates (r): bold indicates significance with a P < 0.05. Log_10_ of LDA values: blue = overrepresented in UD communities, red = overrepresented in MD communities.*

**FIGURE 8:**
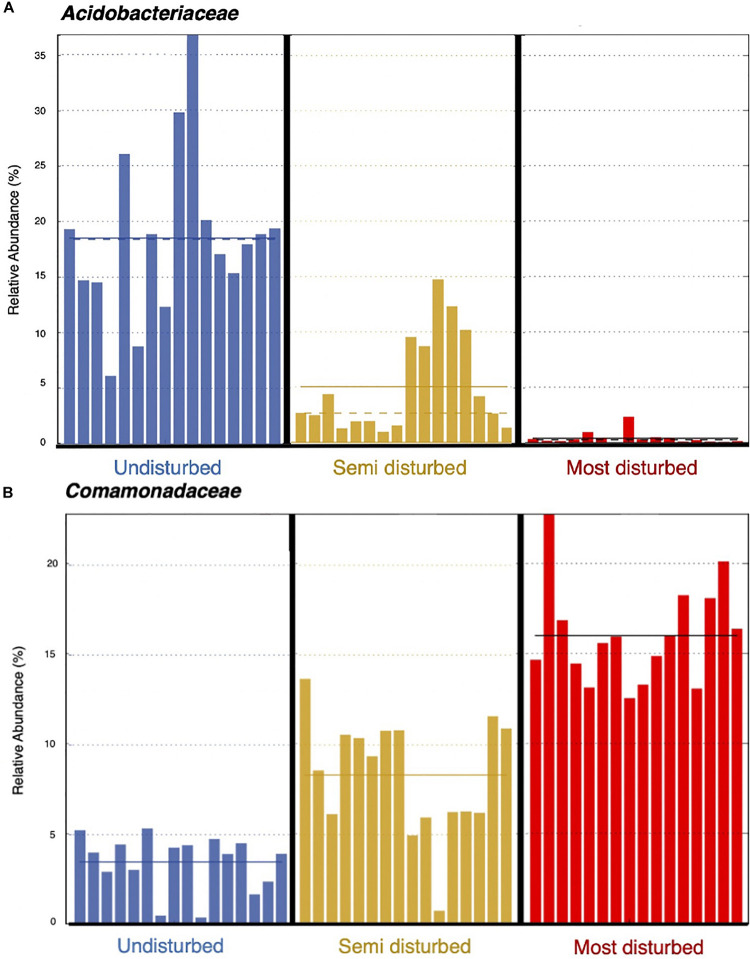
Relative abundances of bacterial families, *Acidobacteriaceae* **(A)** and *Comamonadaceae* **(B)**, per soil core. Solid line represents mean relative abundance per treatment group.

Using the 13 biomarkers that we identified through LEfSe, we completed correlation analyses (Pearson’s correlation coefficient) between the taxonomic biomarkers and plant productivity measures. We found the relative abundance of UD biomarkers to be positively correlated with the height and leaf count for blueberry and cranberry, and with the leaf count of black spruce. One of the UD biomarkers, *Acidobacteriaceae*, showed the strongest positive correlation between growth measures, and was present at a mean relative abundance of 18% in UD communities compared to the SD (5%) and MD (>5%) soil communities (Table 1 and Figure 8A). Of the MD biomarkers, all nine showed significant negative correlations between relative abundance and the productivity measures for blueberry, cranberry, and Labrador tea (Table 1). Looking at the family level, the most negative correlations occurred between plant growth and *Comamonadaceae*, a family that was found to be present at significantly higher relative abundances within the MD soil microbial communities with a mean of 16%, compared to in UD (5%) and SD (8%) soils (Table 1 and Figure 8B). We observed no significant correlations between fireweed plant productivity measures and biomarkers (Table 1).

## Discussion

Our results indicate that there is a strong relationship between microbial community inoculant and total plant growth. We have evidence that microbes associated with highly disturbed soil negatively affect plant productivity within several boreal plant species. Within our study we see similar growth response patterns across low-bush cranberry, bog blueberry, Labrador tea, and fireweed plants. Our analyses also revealed that microbial community membership and abundance shifts across active layer soils above a permafrost thaw gradient. Several studies have also observed soil microbial community responses to permafrost thaw and soil disturbance events (Mackelprang et al., 2011; Coolen and Orsi, 2015; Mondav et al., 2017), and previous research completed by Schütte et al. (2019) has identified links between fungal microbial communities associated with permafrost thaw and plant growth.

Our research showed that plants including bog blueberry, low-bush cranberry, and Labrador tea demonstrated decreased height and leaf count when grown with microbial communities from highly disturbed soil that was associated with deep permafrost thaw compared to undisturbed soil associated with little to no permafrost thaw. Fireweed plants did not show any changes in growth dependent on soil inoculant when looking at leaf count and height. However, when looking at above-ground biomass, plants inoculated with MD soil communities did exhibit lower levels of growth compared to plants inoculated with UD or SD soils. The evidence of this pattern when looking at biomass could potentially be due to the large variation in leaf area and stem thickness that was not present within other plant species that we grew. Additionally, Fireweed is a robust perennial plant, which is known to tolerate a wide range of soil conditions. It is early successional and very commonly following vegetation disturbance (Dyrness, 1973; Pinno et al., 2014). Due to these factors, we speculated that fireweed would not be affected by the disturbance level of the inoculum.

We can attribute the changes in plant growth to the variation in microbial communities across the active layer of soil above the permafrost thaw gradient. We hypothesized that if the soil microbial communities were to change depending on the level of associated permafrost thaw, so would the growth of plants in that soil. Consistent with our hypothesis, plants (specifically bog blueberry, low-bush cranberry, Labrador tea, and fireweed) grown with microbial inoculum from MD soils exhibited lower growth than plants grown in soils with no added microbes or with inoculants from the UD or SD soils. These results were similar to a study performed by Schütte et al. (2019) on boreal plant species using microbial inoculums from thermokarst bogs (disturbed soils) and permafrost plateaus (undisturbed soil). Schütte et al. (2019) showed that bog blueberry and marsh-cinquefoil (*Potentilla palustris*) grew significantly worse when inoculated with soil microbes from thermokarst bogs than when inoculated by permafrost plateau soil microbes.

We found no apparent relationship between black spruce growth and the initial soil microbial inoculant. While Schütte et al. (2019) found that biomass decreased black spruce when grown with inoculum from a thawed permafrost site, our results are consistent with Sniderhan and Baltzer (2016) analyzing the growth of black spruce across a lateral permafrost thaw gradient in Scotty Creek, Canada. They found that the lateral thaw rate of permafrost did not appear to be a driver of black spruce growth dynamics. Further, controlled warming experiments meant to simulate the quickly warming northern latitudes have shown that black spruce shoot length tends to increase with warming air and soil temperatures (Bronson et al., 2009; Bronson and Gower, 2010). This suggests that while productivity of black spruce may be decreased in some thaw scenarios, overall abiotic factors may be having larger influences on black spruce productivity compared to biotic factors during climate warming events.

Previous studies using both culture-based and culture-independent approaches have been able to identify significant functional and taxonomic shifts in soil microbiomes resulting from various types of disturbance (Axelrood et al., 2002; Mendes et al., 2015; Van Nuland et al., 2020). Variation in plant growth across treatments could largely be due to a change in representation from growth-promoting bacterial and fungal species to deleterious, pathogenic ones found in the soil microbiomes between treatment groups. Beneficial microbes in soil are known to enhance nutrient availability to plants, allowing for an increase in plant growth and productivity (Vimal et al., 2017). Common indicators of a neutral or healthy soil microbiome include wide array of bacterial phyla such as *Proteobacteria, Acidobacteria, Actinobacteria*, and *Bacteriodetes*, as well as and fungal phyla such as *Glomeromycota* (Fierer et al., 2005; Ohsowski et al., 2014; Mendes et al., 2015). Consistent with such a roll, and perhaps the most striking difference in microbial taxa between treatment groups, was the bacterial family *Acidobactericae*, which was largely overrepresented in UD soil communities. *Acidobacteriaceae* was also positively correlated with plant productivity measures of both blueberry and cranberry plants, two plants that showed significantly different growth responses between the different inoculant groups. Members of the *Acidobacteriacae* are thought to be plant-promoters and degrade a variety of simple carbon compounds, such as those that are found in root exudates, which contribute to nutrient cycling and the support of a healthy rhizosphere (Campbell et al., 2010; Kielak et al., 2016). Other groups of bacteria that were found to be present at higher relative abundances in the UD soil cores compared to MD soils, including *Bacillales* which can form endospores and provide protection against and *Enterobacterales*, are both ubiquitous in soil microbiomes.

In contrast, the MD soil cores displayed a much higher relative abundance of bacteria from the family *Comamonadaceae*, a member of *Proteobacteria*, than either the UD or SD cores. Additionally, *Comamonadaceae* showed the strongest negative correlation with plant productivity of all identified biomarkers, indicating that members of this family may be driving the decrease in growth that we observed with blueberry, cranberry, and Labrador tea plants. Members of the family *Comamonadaceae* are known to exhibit pathogenic effects on a variety of plants, such as causing bacterial fruit blotch disease in agricultural settings (Willems et al., 1991; Schaad et al., 2008; Burdman and Walcott, 2012). van der Voort et al. (2016) and Yang et al. (2020) have also showed that soils stressed by drought and heat disturbance, respectively, were found to have overrepresented populations of known plant pathogens including members of *Comamonadaceae* and *Erwiniaceae.* Although few studies have looked at the effects of physical disturbances on microbiomes specifically in arctic or subarctic soils, in previous research we cultured and isolated bacterial strains belonging to *Erwiniaceae* from the FPES MD soil plot (Humphrey et al., 2019). It is possible that the higher presence of bacterial groups including known plant-pathogenic bacteria found in the MD soil microbiomes is leading to the decrease in associated plant growth, caused by a disruption in nutrient cycling and direct alterations to the plant rhizospheres.

Our results suggest that a decrease in plant growth can be linked to changes in the taxonomic makeup of microbial communities. These observed patterns are consistent with Schütte et al. (2019) and underline the importance of understanding plant-soil interactions post disturbance. Data from other disturbance gradients are needed test the generality of these observed patterns. Furthermore, long term studies at sites such as the Alaska Peatland Experiment within the Bonanza Creek LTER demonstrate the effects of permafrost thaw on plant communities, trace gas fluxes, microbial communities, and biogeochemical processes (Mackelprang et al., 2011; Blazewicz et al., 2012; Euskirchen et al., 2014; Klapstein et al., 2014; Hultman et al., 2015; Finger et al., 2016; Neumann et al., 2016; Rupp et al., 2019). It is important that more experimentation integrating microbial mechanisms driving plant growth are needed to understand the patterns we have observed.

Having an increased understanding of how microbial communities that reside above permafrost affect plant growth is important for predicting effects of permafrost thaw on plant communities and ecosystem health. Specifically, for potentially predicting effects on plants, such as bog blueberry, low-bush cranberry, and Labrador tea, that are relied on as common food sources for both humans and animals throughout the Northern regions. Although the links between soil microbial and plant communities are complex and rarely straight-forward, this study indicates that the variation in active layer soil microbiomes can have a strong effect on plant growth. This data point toward building an understanding of how shifting microbial communities may affect plant growth in the face of climate change causing thawing permafrost; specifically, growth of plants such as bog blueberry and low-bush cranberry, both plants integral to the diets and cultures of many people indigenous to Alaska. The results reported here demonstrate that soil microbes have the capability to alter plant growth in response to climate change, therefore highlighting the need for further research on the taxonomy and functionality of microbial communities residing above thawing permafrost.

## Data Availability Statement

The original contributions presented in the study are publicly available. This data can be found here: European Nucleotide Archive (ENA) under study accession number PRJEB42020.

## Author Contributions

US contributed to the concept, design, and analysis of the study. TS performed the data collection and statistical analysis and wrote the first draft of the manuscript. DD was involved in experimental design, data collection, bioinformatic analysis, and drafting the manuscript. All authors contributed to the manuscript revisions and approved the submitted version.

## Conflict of Interest

The authors declare that the research was conducted in the absence of any commercial or financial relationships that could be construed as a potential conflict of interest.

## Footnotes

1 https://github.com/rrwick/Filtlong

2 https://galaxyproject.org/

3 https://huttenhower.sph.harvard.edu/galaxy/
