# Supplementary material for: Soil Disturbance Affects Plant Productivity via Soil Microbial Community Shifts
Source: Front Microbiol. 2021 Feb 1;12:619711. doi: 10.3389/fmicb.2021.619711 (PMC7882522; doi:10.3389/fmicb.2021.619711)
Supplement: Supplementary file 1 [file Table_1.docx]

**Supplementary File**

## Supplementary Table 1. Base calling specifications per sequencing run

| **Run** | **Flow cell + kit model** |
| --- | --- |
| 20180611 | dna_r9.5_450bps |
| 20180719A | dna_r9.4.1_450bps_hac |
| 20180719B | dna_r9.4.1_450bps_hac |
| 20180719C | dna_r9.4.1_450bps_hac |
