# Supplementary material for: Soil Disturbance Affects Plant Productivity via Soil Microbial Community Shifts
Source: Front Microbiol. 2021 Feb 1;12:619711. doi: 10.3389/fmicb.2021.619711 (PMC7882522; doi:10.3389/fmicb.2021.619711)
Supplement: Supplementary file 2 [file Table_2.docx]

**Supplementary File**

## Supplementary Table 2. Summary of sequencing statistics per sample following quality control

| Sample | Run | Barcode | Total yield (bp) | Total read count (bp) | Avg read length (bp) |
| --- | --- | --- | --- | --- | --- |
| MD 9.1 | FPES_20180719A | 05 | 400,202,630 | 194,937 | 2052.98 |
| MD 9.2 | FPES_20180719B | 06 | 309,392,310 | 107,312 | 2883.11 |
| MD 9.3 | FPES_20180611 | 09 | 203,489,778 | 93,380 | 2179.16 |
| MD 9.4 | FPES_20180719C | 05 | 373,793,563 | 140,143 | 2667.23 |
| MD 10.1 | FPES_20180719A | 08 | 335,540,391 | 107,848 | 3111.23 |
| MD 10.2 | FPES_20180611 | 10 | 237,477,806 | 66,156 | 3589.66 |
| MD 10.3 | FPES_20180719B | 07 | 266,280,833 | 95,477 | 2788.95 |
| MD 10.4 | FPES_20180719C | 06 | 528,466,953 | 199,447 | 2649.66 |
| MD 11.1 | FPES_20180611 | 11 | 339,699,261 | 141,202 | 2405.77 |
| MD 11.2 | FPES_20180719A | 07 | 319,858,083 | 151,923 | 2105.4 |
| MD 11.3 | FPES_20180719B | 05 | 244,526,616 | 87,798 | 2785.1 |
| MD 11.4 | FPES_20180719C | 03 | 464,942,414 | 152,761 | 3043.59 |
| MD 12.1 | FPES_20180719A | 01 | 242,429,913 | 104,932 | 2310.35 |
| MD 12.2 | FPES_20180611 | 12 | 187,202,338 | 87,766 | 2132.97 |
| MD 12.3 | FPES_20180719B | 04 | 237,416,454 | 134,994 | 1758.72 |
| MD 12.4 | FPES_20180719C | 02 | 626,845,953 | 198,765 | 3153.7 |
| SD 9.1 | FPES_20180719A | 04 | 683,763,421 | 392,010 | 1744.25 |
| SD 9.2 | FPES_20180719B | 01 | 400,235,372 | 173,176 | 2311.15 |
| SD 9.3 | FPES_20180719C | 07 | 737,067,162 | 299,103 | 2464.26 |
| SD 9.4 | FPES_20180611 | 05 | 203,634,783 | 77,269 | 2635.4 |
| SD 10.1 | FPES_20180719A | 03 | 435,414,246 | 203,659 | 2137.96 |
| SD 10.2 | FPES_20180719B | 03 | 308,506,891 | 136,862 | 2254.15 |
| SD 10.3 | FPES_20180611 | 06 | 304,500,863 | 101,986 | 2985.71 |
| SD 10.4 | FPES_20180719C | 12 | 465,408,883 | 140,711 | 3307.55 |
| SD 11.1 | FPES_20180719A | 02 | 269,690,205 | 129,469 | 2083.05 |
| SD 11.2 | FPES_20180611 | 07 | 182,991,407 | 53,543 | 3417.65 |
| SD 11.3 | FPES_20180719B | 12 | 264,072,945 | 100,301 | 2632.8 |
| SD 11.4 | FPES_20180719C | 10 | 651,807,046 | 220,446 | 2956.77 |
| SD 12.1 | FPES_20180719A | 06 | 523,124,325 | 263,679 | 1983.94 |
| SD 12.2 | FPES_20180611 | 08 | 345,914,971 | 169,926 | 2035.68 |
| SD 12.3 | FPES_20180719B | 02 | 486,981,032 | 246,027 | 1979.38 |
| SD 12.4 | FPES_20180719C | 08 | 699,809,340 | 184,567 | 3791.63 |
| UD 9.1 | FPES_20180611 | 01 | 235,098,257 | 80,943 | 2904.49 |
| UD 9.2 | FPES_20180719A | 09 | 335,750,695 | 122,957 | 2730.64 |
| UD 9.3 | FPES_20180719B | 09 | 316,380,483 | 134,144 | 2358.51 |
| UD 9.4 | FPES_20180719C | 01 | 450,701,122 | 147,992 | 3045.44 |
| UD 10.1 | FPES_20180719A | 10 | 385,328,155 | 124,052 | 3106.18 |
| UD 10.2 | FPES_20180611 | 02 | 275,828,426 | 78,460 | 3515.53 |
| UD 10.3 | FPES_20180719B | 08 | 296,873,474 | 106,709 | 2782.08 |
| UD 10.4 | FPES_20180719C | 04 | 556,101,883 | 178,135 | 3121.8 |
| UD 11.1 | FPES_20180611 | 03 | 295,277,023 | 97,759 | 3020.46 |
| UD 11.2 | FPES_20180719A | 11 | 344,805,302 | 116,453 | 2960.9 |
| UD 11.3 | FPES_20180719B | 11 | 206,576,878 | 101,380 | 2037.65 |
| UD 11.4 | FPES_20180719C | 11 | 516,115,731 | 135,427 | 3811.03 |
| UD 12.1 | FPES_20180719A | 12 | 272,044,502 | 108,655 | 2503.75 |
| UD 12.2 | FPES_20180611 | 04 | 316,589,380 | 90,290 | 3506.36 |
| UD 12.3 | FPES_20180719B | 10 | 293,661,051 | 121,689 | 2413.21 |
| UD 12.4 | FPES_20180719C | 09 | 546,467,017 | 207,151 | 2638.01 |
