# Supplementary material for: Soil Disturbance Affects Plant Productivity via Soil Microbial Community Shifts
Source: Front Microbiol. 2021 Feb 1;12:619711. doi: 10.3389/fmicb.2021.619711 (PMC7882522; doi:10.3389/fmicb.2021.619711)
Supplement: Supplementary file 3 [file Table_3.docx]

**Supplementary File**

## Supplementary Table 3. ANOVA results for bog blueberry growth measures.

| **Growth Measure** | **Source** | **Degrees of freedom** | **Sum of squares** | **Mean sum of squares** | **F value** | **P value** |
| --- | --- | --- | --- | --- | --- | --- |
| **Height** | FPES | 3 | 52965 | 17655 | 11.44 | **5.88 x 10^-6^** |
|  | Residuals | 56 | 86395 | 1543 |  |  |
| **Leaf Count** | FPES | 3 | 16568 | 5523 | 5.544 | **0.00207** |
|  | Residuals | 57 | 56778 | 996 |  |  |
| **Above Ground Biomass** | FPES | 3 | 0.8878 | 0.2959 | 6.663 | **0.000787** |
|  | Residuals | 46 | 2.043 | 0.04441 |  |  |
| **Below Ground Biomass** | FPES | 3 | 0.926 | 0.30862 | 4.189 | **0.0106** |
|  | Residuals | 46 | 3.389 | 0.07367 |  |  |

## *Bolded p-value indicates significance with a < 0.05
