# Supplementary material for: Soil Disturbance Affects Plant Productivity via Soil Microbial Community Shifts
Source: Front Microbiol. 2021 Feb 1;12:619711. doi: 10.3389/fmicb.2021.619711 (PMC7882522; doi:10.3389/fmicb.2021.619711)
Supplement: Supplementary file 4 [file Table_4.docx]

**Supplementary File**

## Supplementary Table 4. ANOVA results for low-bush cranberry growth measures.

| **Growth Measure** | **Response** | **Degrees of freedom** | **Sum of squares** | **Mean sum of squares** | **F value** | **P value** |
| --- | --- | --- | --- | --- | --- | --- |
| **Height** | FPES | 3 | 13881 | 4627 | 8.966 | **6.25 x 10^-5^** |
|  | Residuals | 55 | 28385 | 516 |  |  |
| **Leaf Count** | FPES | 3 | 6502 | 2167.3 | 9.295 | **4.39 x 10^-5^** |
|  | Residuals | 56 | 13057 | 233.2 |  |  |
| **Above Ground Biomass** | FPES | 3 | 1.852 | 0.6173 | 12.98 | **2.32 x 10^-6^** |
|  | Residuals | 49 | 2.331 | 0.0476 |  |  |
| **Below Ground Biomass** | FPES | 3 | 0.525 | 0.17516 | 2.019 | 0.124 |
|  | Residuals | 49 | 4.252 | 0.08677 |  |  |

## *Bolded p-value indicates significance with a < 0.05
