# Supplementary material for: Soil Disturbance Affects Plant Productivity via Soil Microbial Community Shifts
Source: Front Microbiol. 2021 Feb 1;12:619711. doi: 10.3389/fmicb.2021.619711 (PMC7882522; doi:10.3389/fmicb.2021.619711)
Supplement: Supplementary file 5 [file Table_5.docx]

**Supplementary File**

## Supplementary Table 5. ANOVA results for Labrador tea growth measures.

| **Growth Measure** | **Response** | **Degrees of freedom** | **Sum of squares** | **Mean sum of squares** | **F value** | **P value** |
| --- | --- | --- | --- | --- | --- | --- |
| **Height** | FPES | 3 | 12314 | 4105 | 7.947 | **0.000233** |
|  | Residuals | 45 | 23243 | 517 |  |  |
| **Leaf Count** | FPES | 3 | 3476 | 1158.7 | 5.216 | **0.00355** |
|  | Residuals | 45 | 9995 | 222.1 |  |  |
| **Above Ground Biomass** | FPES | 3 | 0.8639 | 0.28795 | 5.65 | **0.00273** |
|  | Residuals | 37 | 1.8858 | 0.0509 |  |  |
| **Below Ground Biomass** | FPES | 3 | 6.6 | 2.1994 | 2.377 | 0.0855 |
|  | Residuals | 37 | 34.23 | 0.9251 |  |  |

## *Bolded p-value indicates significance with a < 0.05
