# Supplementary material for: Soil Disturbance Affects Plant Productivity via Soil Microbial Community Shifts
Source: Front Microbiol. 2021 Feb 1;12:619711. doi: 10.3389/fmicb.2021.619711 (PMC7882522; doi:10.3389/fmicb.2021.619711)
Supplement: Supplementary file 6 [file Table_6.docx]

**Supplementary File**

## Supplementary Table 6. ANOVA results for fireweed growth measures.

| **Growth Measure** | **Response** | **Degrees of freedom** | **Sum of squares** | **Mean sum of squares** | **F value** | **P value** |
| --- | --- | --- | --- | --- | --- | --- |
| **Height** | FPES | 3 | 13439 | 4480 | 3.053 | **0.0356** |
|  | Residuals | 57 | 83628 | 1467 |  |  |
| **Leaf Count** | FPES | 3 | 0.1567 | 0.05224 | 1.447 | 0.239 |
|  | Residuals | 55 | 1.9858 | 0.0361 |  |  |
| **Above Ground Biomass** | FPES | 3 | 1.51 | 0.5034 | 11.17 | **7.24 x 10^-6^** |
|  | Residuals | 57 | 2.57 | 0.0451 |  |  |

## *Bolded p-value indicates significance with a < 0.05
