# Supplementary material for: Soil Disturbance Affects Plant Productivity via Soil Microbial Community Shifts
Source: Front Microbiol. 2021 Feb 1;12:619711. doi: 10.3389/fmicb.2021.619711 (PMC7882522; doi:10.3389/fmicb.2021.619711)
Supplement: Supplementary file 7 [file Table_7.docx]

**Supplementary File**

## Supplementary Table 7. ANOVA results for black spruce growth measures.

| **Growth Measure** | **Response** | **Degrees of freedom** | **Sum of squares** | **Mean sum of squares** | **F value** | **P value** |
| --- | --- | --- | --- | --- | --- | --- |
| **Height** | FPES | 3 | 3634 | 1211.3 | 5.654 | **0.00186** |
|  | Residuals | 57 | 12231 | 214.6 |  |  |
| **Leaf Count** | FPES | 3 | 1.292 | 0.4307 | 2.378 | 0.0787 |
|  | Residuals | 60 | 10.869 | 0.1811 |  |  |
| **Above Ground Biomass** | FPES | 3 | 0.871 | 0.29023 | 4.154 | **0.0101** |
|  | Residuals | 55 | 3.843 | 0.06987 |  |  |
| **Below Ground Biomass** | FPES | 3 | 9.71 | 3.236 | 4.264 | **0.00896** |
|  | Residuals | 54 | 40.99 | 0.759 |  |  |

## *Bolded p-value indicates significance with a < 0.05
