# Supplementary material for: Soil Disturbance Affects Plant Productivity via Soil Microbial Community Shifts
Source: Front Microbiol. 2021 Feb 1;12:619711. doi: 10.3389/fmicb.2021.619711 (PMC7882522; doi:10.3389/fmicb.2021.619711)
Supplement: Supplementary file 8 [file Table_8.docx]

**Supplementary File**

## Supplementary Table 8. Tukey’s honestly significant difference (HSD) post-hoc test results per growth measure and plant type

|  | Growth Measure | UD | SD | MD | ST |
| --- | --- | --- | --- | --- | --- |
| Blueberry | Height | a | a | b | a |
|  | Leaf Count | a | a | b | a |
|  | Above Ground Biomass | ab | a | b | b |
|  | Below Ground Biomass | ab | a | ab | b |
| Cranberry | Height | a | a | b | a |
|  | Leaf Count | a | a | b | a |
|  | Above Ground Biomass | b | a | b | b |
|  | Below Ground Biomass | a | a | a | a |
| Labrador tea | Height | a | a | b | a |
|  | Leaf Count | a | a | b | a |
|  | Above Ground Biomass | a | a | b | ab |
|  | Below Ground Biomass | a | a | a | a |
| Fireweed | Height | ab | a | ab | b |
|  | Leaf Count | a | a | a | a |
|  | Above Ground Biomass | a | a | b | a |
| Black spruce | Height | b | ab | b | a |
|  | Leaf Count | a | a | a | a |
|  | Above Ground Biomass | ab | a | ab | b |
|  | Below Ground Biomass | ab | a | ab | b |
|  |  |  |  |  |  |
