# Supplementary material for: Soil Disturbance Affects Plant Productivity via Soil Microbial Community Shifts
Source: Front Microbiol. 2021 Feb 1;12:619711. doi: 10.3389/fmicb.2021.619711 (PMC7882522; doi:10.3389/fmicb.2021.619711)
Supplement: Supplementary file 9 [file Table_9.docx]

**Supplementary File**

## Supplementary Table 9. Plant productivity measurements per plant from Figures 2-6

| **FPES** | **Species** | **Label** | **Height (mm)** | **Leaf Count** | **AG mass (g)** | **BG mass (g)** |
| --- | --- | --- | --- | --- | --- | --- |
| MD | BB | AA011 | 146 | 111 | 0.7876 | 1.4819 |
| ST | BB | AA018 | 151 | 46 | 0.6642 | 0.2699 |
| ST | BB | AA021 | 78 | 33 | 0.1947 | 0.6521 |
| SD | BB | AA053 | 114 | 78 | 1.0644 | 0.973 |
| SD | BB | AA077 | 119 | 61 | 0.5258 | 0.5472 |
| MD | BB | AA078 | 60 | 17 | 0.1962 | 0.2483 |
| ST | BB | AA083 | 147 | 59 | 0.4423 | 0.3989 |
| ST | BB | AA084 | 197 | 61 | 0.3551 | 0.1076 |
| MD | BB | AA087 | 79 | 34 | 0.2376 | 0.6054 |
| ST | BB | AA090 | 108 | 61 | 0.4998 | 0.545 |
| SD | BB | AA092 | 23 | 18 | 0.1169 |  |
| SD | BB | AA110 | 159 | 81 | 0.5984 | 1.1048 |
| UD | BB | AA113 | 157 | 56 | 0.5863 | 0.8748 |
| MD | BB | AA114 | 10 | 4 |  |  |
| ST | BB | AA120 | 96 | 80 | 0.5895 | 0.6584 |
| UD | BB | AA121 | 125 | 43 | 0.5194 | 1.3794 |
| MD | BB | AA129 | 15 | 11 |  |  |
| UD | BB | AA143 | 162 | 44 | 0.6985 | 0.4929 |
| SD | BB | AA166 | 173 | 57 | 0.9076 | 0.8787 |
| UD | BB | AA174 | 164 | 50 | 1.1324 | 0.7204 |
| SD | BB | AA175 | 121 | 82 | 0.6602 | 0.4306 |
| SD | BB | AA176 | 143 | 71 | 0.7074 | 0.9825 |
| UD | BB | AA189 | 102 | 103 | 0.3216 | 0.8223 |
| UD | BB | AA196 | 164 | 81 | 0.6309 | 0.4621 |
| MD | BB | AA199 | 63 | 46 | 0.4728 | 0.5418 |
| ST | BB | AA200 | 135 | 89 | 0.3417 | 0.7141 |
| UD | BB | AA204 | 109 | 54 | 0.3005 | 0.3724 |
| UD | BB | AA208 | 129 | 53 | 0.1938 | 0.0556 |
| ST | BB | AA212 | 114 | 59 | 0.19 | 0.0941 |
| MD | BB | AA219 | 51 | 37 | 0.5667 | 0.8891 |
| SD | BB | AA221 | 162 | 85 | 0.7894 | 0.9189 |
| UD | BB | AA226 | 168 | 81 | 0.9542 | 0.5081 |
| MD | BB | AA232 | 7 | 7 |  |  |
| MD | BB | AA238 | 104 | 41 | 0.4159 | 0.4667 |
| ST | BB | AA243 | 101 | 49 | 0.4125 | 0.4716 |
| UD | BB | AA250 | 120 | 99 | 1.1085 | 1.0713 |
| ST | BB | AA266 | 112 | 88 | 0.6547 | 0.6531 |
| UD | BB | AA277 | 114 | 83 | 0.6812 | 0.8287 |
| ST | BB | AA283 | 165 | 132 | 0.437 | 0.4782 |
| MD | BB | AA286 | 56 | 33 | 0.3192 | 0.4133 |
| ST | BB | AA290 | 223 | 174 | 0.1739 | 0.043 |
| SD | BB | AA295 | 111 | 117 | 0.8647 | 1.1165 |
| SD | BB | AA296 | 80 | 37 | 0.2557 |  |
| ST | BB | AA297 | 115 | 65 | 0.7504 | 0.6292 |
| SD | BB | AA303 | 122 | 89 | 0.7013 | 0.8376 |
| SD | BB | AA311 | 100 | 31 | 0.4894 | 0.8038 |
| SD | BB | AA312 | 141 | 99 | 0.5468 | 0.6055 |
| SD | BB | AA315 | 101 | 66 | 0.8143 | 0.5709 |
| UD | BB | AA319 | 159 | 179 | 0.6496 | 0.93 |
| ST | BB | AA321 | 83 | 40 | 0.3912 | 0.5965 |
| MD | BB | AA333 | 29 | 45 | 0.3067 | 0.322 |
| SD | BB | AA341 | 189 | 62 | 0.5348 | 0.5618 |
| ST | BB | AA347 | 57 | 49 | 0.3849 | 0.3185 |
| MD | BB | AA357 | 122 | 44 | 0.5784 | 0.4786 |
| SD | BB | AA358 | 153 | 52 | 0.8462 | 0.4564 |
| UD | BB | AA360 | 52 | 93 | 0.3444 | 0.1429 |
| MD | BB | AA364 | 13 | 7 | 0.398 | 0.3512 |
| UD | BB | AA368 | 191 | 65 | 0.6317 | 0.9285 |
| MD | BB | AA372 | 95 | 53 | 0.4493 | 0.6264 |
| MD | BB | AA374 | 111 | 50 | 0.4902 | 0.6891 |
| UD | BB | AA382 | 151 | 106 | 0.4696 | 0.3941 |
| UD | CB | AA002 | 68 | 57 | 0.2866 | 0.5829 |
| UD | CB | AA010 | 38 | 17 | 0.39 | 0.4937 |
| ST | CB | AA027 | 46 | 20 | 0.3593 | 0.4543 |
| ST | CB | AA034 | 56 | 47 | 0.6274 | 0.7143 |
| UD | CB | AA037 | 75 | 20 | 0.8557 | 0.9865 |
| MD | CB | AA041 | 13 | 18 | 0.5245 | 0.9524 |
| ST | CB | AA046 | 73 | 58 | 0.5109 | 0.7088 |
| MD | CB | AA048 | 67 | 20 | 0.8839 | 0.6945 |
| ST | CB | AA061 | 56 | 37 | 0.3323 | 0.2136 |
| MD | CB | AA065 | 45 | 23 | 0.6069 | 0.6088 |
| ST | CB | AA070 | 63 | 43 | 0.3795 | 0.1809 |
| SD | CB | AA081 | 36 | 11 | 0.804 | 0.1916 |
| UD | CB | AA089 | 58 | 56 | 0.8361 | 0.3655 |
| ST | CB | AA094 | 64 | 38 | 0.3559 | 0.275 |
| SD | CB | AA116 | 85 | 37 | 0.8462 | 0.3869 |
| ST | CB | AA117 | 16 | 27 | 0.2068 | 0.2712 |
| SD | CB | AA131 | 42 | 28 | 0.9662 | 0.5885 |
| ST | CB | AA133 | 20 | 11 | 0.4041 | 0.7771 |
| SD | CB | AA135 | 82 | 70 | 1.0738 | 1.0907 |
| MD | CB | AA141 | 8 | 3 | 0.1272 | 0.5264 |
| UD | CB | AA145 | 60 | 33 | 0.4797 | 0.6003 |
| SD | CB | AA146 | 90 | 33 | 1.0027 | 0.3351 |
| ST | CB | AA148 | 50 | 42 | 0.3004 | 0.5484 |
| SD | CB | AA151 | 30 | 24 | 0.8075 | 0.812 |
| UD | CB | AA152 | 75 | 32 | 0.8822 | 0.6276 |
| SD | CB | AA154 | 100 | 26 | 1.1253 | 0.5024 |
| SD | CB | AA177 | 70 | 53 | 0.5864 | 1.8036 |
| MD | CB | AA178 | 10 | 4 |  |  |
| SD | CB | AA182 | 52 | 58 | 1.0169 | 1.069 |
| SD | CB | AA188 | 53 | 36 | 0.6966 | 0.5734 |
| UD | CB | AA201 | 72 | 83 | 0.6983 | 1.007 |
| ST | CB | AA202 | 71 | 44 | 0.2273 | 0.5293 |
| MD | CB | AA203 | 9 | 6 | 0.1565 | 0.2093 |
| UD | CB | AA216 | 90 | 41 | 0.2484 |  |
| MD | CB | AA223 | 23 | 16 | 0.8322 | 0.7875 |
| UD | CB | AA235 | 72 | 28 | 0.3902 | 0.293 |
| MD | CB | AA237 | 19 | 11 | 0.4135 | 0.483 |
| ST | CB | AA264 | 16 | 5 | 0.5944 | 0.255 |
| MD | CB | AA269 | 48 | 26 | 0.9528 | 0.4994 |
| SD | CB | AA276 | 18 | 57 | 0.7095 | 0.7574 |
| MD | CB | AA291 | 4 | 4 | 0.126 | 0.4079 |
| UD | CB | AA292 | 7 | 9 |  |  |
| SD | CB | AA294 | 30 | 38 | 0.6238 | 0.6342 |
| UD | CB | AA305 | 108 | 46 | 0.4092 | 0.5162 |
| ST | CB | AA307 | 20 | 14 | 0.2656 | 0.1558 |
| ST | CB | AA320 | 48 | 23 | 0.4687 | 0.224 |
| UD | CB | AA323 | 55 | 28 | 0.565 | 0.3825 |
| UD | CB | AA332 | 66 | 51 | 0.6046 | 0.3454 |
| SD | CB | AA334 | 33 | 26 | 0.8161 | 0.4205 |
| SD | CB | AA335 | 43 | 36 | 0.7042 | 0.4033 |
| SD | CB | AA342 | 67 | 54 | 0.7321 | 0.4955 |
| UD | CB | AA346 | 55 | 41 | 0.6316 | 0.7428 |
| ST | CB | AA348 | 47 | 42 | 0.5027 | 0.6528 |
| SD | CB | AA349 | 72 | 20 | 1.2656 | 0.8958 |
| MD | CB | AA352 | 4 | 6 | 0.0345 | 0.0257 |
| ST | CB | AA355 | 32 | 21 | 0.5136 | 0.4826 |
| MD | CB | AA365 | 5 | 4 | 0.0524 | 0.0313 |
| MD | CB | AA369 | 5 | 6 | 0.0092 | 0.0076 |
| MD | CB | AA371 | 56 | 25 | 0.684 | 0.1712 |
| ST | CB | AA378 | 78 | 37 | 0.6585 | 0.5887 |
| MD | LT | AA013 | 3 | 7 | 0.2654 | 0.4162 |
| ST | LT | AA025 | 50 | 40 | 0.113 |  |
| SD | LT | AA030 | 67 | 44 | 0.8583 | 1.3481 |
| ST | LT | AA035 | 73 | 62 | 0.4782 | 2.0208 |
| UD | LT | AA047 | 54 | 34 |  |  |
| MD | LT | AA055 | 12 | 13 | 0.0673 | 0.0692 |
| MD | LT | AA057 | 2 | 6 | 0.1871 | 0.3126 |
| SD | LT | AA063 | 40 | 32 | 0.5846 | 0.3423 |
| SD | LT | AA086 | 85 | 60 | 0.7004 | 0.35523 |
| SD | LT | AA095 | 60 | 37 | 0.5506 | 0.808 |
| SD | LT | AA106 | 46 | 25 | 0.435 | 0.9877 |
| UD | LT | AA109 | 77 | 37 | 0.7343 | 1.9727 |
| ST | LT | AA111 | 40 | 41 | 0.3771 | 0.4716 |
| ST | LT | AA122 | 64 | 39 | 0.5327 | 0.6236 |
| SD | LT | AA124 | 35 | 32 | 0.2929 | 0.1549 |
| ST | LT | AA126 | 33 | 19 | 0.2754 | 0.7225 |
| MD | LT | AA139 | 2 | 3 | 0.3349 | 0.2019 |
| UD | LT | AA147 | 41 | 48 | 0.548 | 0.7748 |
| MD | LT | AA164 | 3 | 5 | 0.0328 | 0.0115 |
| ST | LT | AA173 | 31 | 60 | 0.6219 | 1.0088 |
| SD | LT | AA193 | 32 | 17 | 0.7655 | 0.5727 |
| MD | LT | AA197 | 27 | 18 | 0.477 | 0.7145 |
| UD | LT | AA215 | 73 | 45 | 0.5404 | 1.7485 |
| SD | LT | AA222 | 15 | 26 | 0.988 | 0.4322 |
| UD | LT | AA225 | 80 | 25 | 0.4738 | 0.4251 |
| ST | LT | AA228 | 5 | 7 |  |  |
| MD | LT | AA229 | 11 | 18 | 0.1878 | 0.2865 |
| UD | LT | AA244 | 1 | 1 | 0.0193 |  |
| ST | LT | AA252 | 25 | 19 | 0.3069 | 0.4641 |
| UD | LT | AA254 | 1 | 0.01 |  |  |
| MD | LT | AA256 | 4 | 6 | 0.3061 | 0.4705 |
| UD | LT | AA265 | 75 | 48 | 0.921 | 0.4114 |
| SD | LT | AA270 | 30 | 12 | 0.0416 |  |
| MD | LT | AA275 | 3 | 6 |  |  |
| ST | LT | AA284 | 9 | 11 | 0.0283 |  |
| ST | LT | AA298 | 10 | 11 | 0.1234 | 0.3279 |
| SD | LT | AA300 | 45 | 21 | 0.4653 | 0.6575 |
| ST | LT | AA306 | 12 | 11 | 0.1372 | 0.4592 |
| UD | LT | AA314 | 16 | 18 | 0.3586 | 0.12278 |
| UD | LT | AA322 | 50 | 26 | 0.4355 | 0.2982 |
| SD | LT | AA325 | 85 | 35 | 1.0974 | 0.8364 |
| UD | LT | AA326 | 68 | 32 | 0.282 | 0.4038 |
| ST | LT | AA329 | 62 | 32 | 0.8544 | 0.5958 |
| SD | LT | AA340 | 54 | 46 | 0.124 | 0.0385 |
| SD | LT | AA354 | 52 | 26 | 0.5726 | 1.5944 |
| UD | LT | AA356 | 72 | 52 | 0.8205 | 0.2121 |
| ST | LT | AA359 | 43 | 36 | 0.3195 | 0.2125 |
| SD | LT | AA363 | 17 | 21 | 0.3986 |  |
| SD | LT | AA375 | 75 | 43 | 0.1938 | 0.0760 |
| UD | FW | AA016 | 57 | 18 | 0.1613 |  |
| UD | FW | AA017 | 101 | 15 | 0.2097 |  |
| MD | FW | AA026 | 95 | 25 | 0.1346 |  |
| UD | FW | AA036 | 64 | 19 | 0.1986 |  |
| SD | FW | AA045 | 81 | 20 | 0.155 |  |
| ST | FW | AA049 | 87 | 20 | 0.193 |  |
| MD | FW | AA052 | 35 | 17 | 0.1384 |  |
| UD | FW | AA054 | 83 | 17 | 0.2145 |  |
| ST | FW | AA058 | 104 | 21 | 0.2711 |  |
| SD | FW | AA060 | 37 | 23 | 0.2462 |  |
| ST | FW | AA067 | 95 | 18 | 0.2034 |  |
| UD | FW | AA069 | 78 | 12 | 0.2839 |  |
| SD | FW | AA071 | 45 | 15 | 0.267 |  |
| ST | FW | AA076 | 96 | 24 | 0.1737 |  |
| UD | FW | AA091 | 76 | 18 | 0.2398 |  |
| ST | FW | AA097 | 80 | 18 | 0.2111 |  |
| ST | FW | AA099 | 125 | 21 | 0.2943 |  |
| MD | FW | AA100 | 94 | 30 | 0.1774 |  |
| ST | FW | AA112 | 70 | 18 | 0.2042 |  |
| UD | FW | AA115 | 111 | 22 | 0.2223 |  |
| ST | FW | AA119 | 129 | 24 | 0.3024 |  |
| SD | FW | AA123 | 110 | 23 | 0.2222 |  |
| SD | FW | AA127 | 69 | 19 | 0.1764 |  |
| MD | FW | AA130 | 72 | 16 | 0.167 |  |
| ST | FW | AA132 | 163 | 34 | 0.286 |  |
| MD | FW | AA134 | 72 | 21 | 0.147 |  |
| MD | FW | AA138 | 114 | 18 | 0.1486 |  |
| SD | FW | AA142 | 192 | 48 | 0.2404 |  |
| UD | FW | AA149 | 95 | 27 | 0.2323 |  |
| SD | FW | AA150 | 111 | 20 | 0.2256 |  |
| SD | FW | AA155 | 78 | 15 | 0.116 |  |
| SD | FW | AA156 | 97 | 20 | 0.2046 |  |
| UD | FW | AA161 | 97 | 19 | 0.2198 |  |
| UD | FW | AA167 | 131 | 22 | 0.275 |  |
| MD | FW | AA170 | 48 | 13 | 0.143 |  |
| MD | FW | AA172 | 65 | 24 | 0.1374 |  |
| UD | FW | AA179 | 103 | 19 | 0.2376 |  |
| SD | FW | AA183 | 171 | 28 | 0.2808 |  |
| ST | FW | AA185 | 59 | 28 | 0.1638 |  |
| SD | FW | AA198 | 72 | 18 | 0.2097 |  |
| MD | FW | AA211 | 43 | 18 | 0.1664 |  |
| UD | FW | AA213 | 96 | 25 | 0.2101 |  |
| MD | FW | AA220 | 66 | 17 | 0.1523 |  |
| MD | FW | AA227 | 70 | 16 | 0.1458 |  |
| ST | FW | AA230 | 113 | 20 | 0.3006 |  |
| ST | FW | AA236 | 91 | 20 | 0.3457 |  |
| MD | FW | AA241 | 88 | 18 | 0.1366 |  |
| MD | FW | AA242 | 102 | 20 | 0.1874 |  |
| SD | FW | AA255 | 122 | 22 | 0.2588 |  |
| UD | FW | AA257 | 112 | 22 | 0.2108 |  |
| MD | FW | AA263 | 78 | 20 | 0.1772 |  |
| MD | FW | AA278 | 107 | 22 | 0.1719 |  |
| SD | FW | AA281 | 95 | 21 | 0.2356 |  |
| UD | FW | AA285 | 115 | 18 | 0.2177 |  |
| ST | FW | AA293 | 97 | 26 | 0.2599 |  |
| MD | FW | AA301 | 98 | 22 | 0.2691 |  |
| SD | FW | AA317 | 153 | 19 | 0.2933 |  |
| ST | FW | AA330 | 101 | 8 | 0.2428 |  |
| ST | FW | AA337 | 105 | 19 | 0.1666 |  |
| SD | FW | AA361 | 57 | 20 | 0.1651 |  |
| ST | FW | AA379 | 138 | 22 | 0.35 |  |
| ST | BS | AA004 | 180 | 359 | 1.1466 | 1.835 |
| UD | BS | AA014 | 86 | 83 | 0.6208 | 1.4985 |
| ST | BS | AA019 | 105 | 226 | 0.5569 | 1.6612 |
| MD | BS | AA033 | 74 | 281 | 1.119 | 2.6954 |
| SD | BS | AA040 | 87 | 112 | 1.47 | 3.794 |
| UD | BS | AA042 | 88 | 132 | 0.7419 | 2.4321 |
| UD | BS | AA051 | 90 | 74 | 0.7422 | 2.0013 |
| ST | BS | AA056 | 115 | 109 | 0.9997 | 2.3505 |
| ST | BS | AA059 | 112 | 153 | 0.8701 | 1.5131 |
| ST | BS | AA062 | 113 | 274 | 0.9233 | 2.592 |
| MD | BS | AA064 | 78 | 209 | 1.2267 | 3.811 |
| MD | BS | AA072 | 82 | 298 | 0.8774 | 1.519 |
| SD | BS | AA075 | 90 | 66 | 0.8624 | 3.8218 |
| ST | BS | AA080 | 96 | 308 | 0.2583 | 0.3933 |
| MD | BS | AA082 | 104 | 125 | 0.6113 | 1.3944 |
| SD | BS | AA098 | 70 | 54 | 1.0323 | 3.1497 |
| ST | BS | AA101 | 110 | 132 | 0.602 | 1.4962 |
| SD | BS | AA118 | 116 | 234 | 1.1038 | 4.2056 |
| SD | BS | AA144 | 120 | 236 | 1.4602 | 2.2203 |
| UD | BS | AA157 | 111 | 153 | 0.9606 | 1.9147 |
| ST | BS | AA158 | 134 | 304 | 0.8833 | 3.6227 |
| MD | BS | AA160 | 90 | 188 | 1.2234 | 4.409 |
| UD | BS | AA162 | 87 | 120 | 0.8193 | 2.3029 |
| MD | BS | AA163 | 92 | 97 | 0.4599 | 2.1533 |
| MD | BS | AA165 | 67 | 98 | 1.1801 | 2.7645 |
| MD | BS | AA168 | 85 | 135 | 0.6503 | 2.8478 |
| SD | BS | AA169 | 83 | 119 | 1.299 | 3.3789 |
| ST | BS | AA171 | 121 | 162 | 1.1103 | 2.786 |
| SD | BS | AA181 | 119 | 177 | 0.904 | 2.3104 |
| UD | BS | AA186 | 84 | 113 | 0.8381 | 3.0546 |
| UD | BS | AA191 | 125 | 156 | 1.0492 | 2.729 |
| SD | BS | AA209 | 89 | 139 | 1.074 | 2.8078 |
| UD | BS | AA210 | 65 | 84 | 0.6737 | 2.1269 |
| SD | BS | AA218 | 95 | 134 | 0.8098 | 2.9974 |
| ST | BS | AA234 | 90 | 93 | 0.6321 | 1.5233 |
| MD | BS | AA245 | 95 | 142 | 1.3896 | 3.5156 |
| UD | BS | AA249 | 64 | 97 | 0.6202 | 2.0843 |
| UD | BS | AA253 | 81 | 99 | 0.7009 | 1.8047 |
| ST | BS | AA260 | 99 | 166 | 0.6836 | 2.4496 |
| UD | BS | AA261 | 74 | 103 | 0.5202 | 1.0629 |
| UD | BS | AA267 | 110 | 188 | 0.8595 | 2.6763 |
| SD | BS | AA268 | 86 | 128 | 0.6417 | 2.2179 |
| SD | BS | AA271 | 90 | 74 | 0.6555 | 1.5805 |
| ST | BS | AA274 | 100 | 181 | 0.4934 | 1.0019 |
| ST | BS | AA279 | 126 | 118 | 0.6533 | 1.2182 |
| SD | BS | AA287 | 91 | 131 | 1.1361 | 3.0523 |
| SD | BS | AA299 | 92 | 124 | 0.9727 | 1.9379 |
| MD | BS | AA302 | 96 | 199 | 0.867 | 1.8782 |
| UD | BS | AA309 | 96 | 241 | 1.2462 | 1.7297 |
| ST | BS | AA310 | 82 | 110 | 0.3959 | 0.5624 |
| UD | BS | AA316 | 66 | 105 | 0.3597 | 1.0634 |
| SD | BS | AA318 | 109 | 141 | 0.9649 | 2.1004 |
| MD | BS | AA327 | 66 | 101 | 0.668 | 1.712 |
| ST | BS | AA336 | 91 | 137 | 0.2807 | 0.3196 |
| SD | BS | AA338 | 94 | 125 | 0.8408 | 1.7795 |
| SD | BS | AA339 | 97 | 184 | 0.6453 | 0.8773 |
| MD | BS | AA350 | 82 | 162 | 0.7026 | 1.6296 |
| MD | BS | AA353 | 96 | 153 | 1.1528 | 2.0911 |
| ST | BS | AA362 | 40 | 35 | 0.6931 | 1.8461 |
| UD | BS | AA367 | 81 | 86 | 0.7595 | 1.5437 |
| UD | BS | AA370 | 87 | 97 | 0.5758 | 1.4991 |
| MD | BS | AA380 | 102 | 226 | 1.1233 | 3.4713 |
| MD | BS | AA381 | 97 | 127 | 1.1793 | 4.2128 |
| MD | BS | AA384 | 102 | 177 | 0.9324 | 0.3704 |

*Note about data collection:*

Height for all plants was compared at day 184, after the growth rate had started to slow (except fireweed which was compared at day 121 due to harvest for biomass). Leaf count was compared at day 184 for BB, CB, and LT after growth had stabilized. Leaf count for fireweed was analyzed on day 121 due to harvest, and black spruce was analyzed on day 121 (the last needle count collected, due to challenges with collection accuracy). Only above ground biomass was collected for fireweed on day 121. Above ground biomass was collected 640 days following planting for all other plant types (BB, CB, BS, and LT). Below ground biomass was collected at a delayed date 665 days after planting, due to complications related to COVID-19. Some plants did not have any discernable biomass during collection, due to senescence or the size of the plant, and those plants have not recorded biomasses (Supplementary Table 9).
