# Supplementary material for: Soil Disturbance Affects Plant Productivity via Soil Microbial Community Shifts
Source: Front Microbiol. 2021 Feb 1;12:619711. doi: 10.3389/fmicb.2021.619711 (PMC7882522; doi:10.3389/fmicb.2021.619711)
Supplement: Supplementary file 10 [file Data_Sheet_1.pdf]

Supplementary File 1

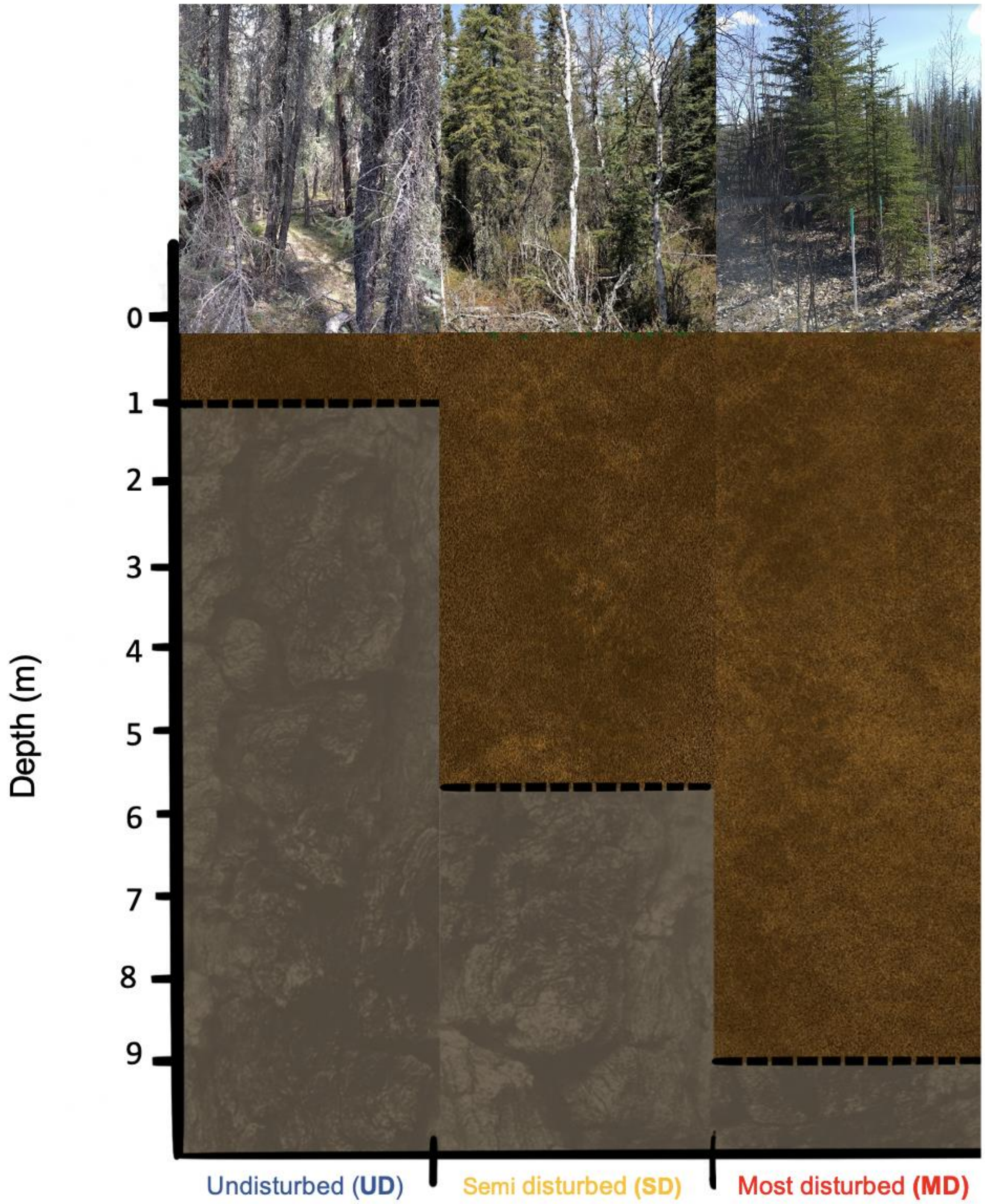

Supplementary Figure 1. Fairbanks Permafrost Experiment Station (FPES) plot layout and permafrost thaw levels and above ground plant cover
