# Supplementary material for: Soil Disturbance Affects Plant Productivity via Soil Microbial Community Shifts
Source: Front Microbiol. 2021 Feb 1;12:619711. doi: 10.3389/fmicb.2021.619711 (PMC7882522; doi:10.3389/fmicb.2021.619711)
Supplement: Supplementary file 11 [file Data_Sheet_2.pdf]

Supplementary File

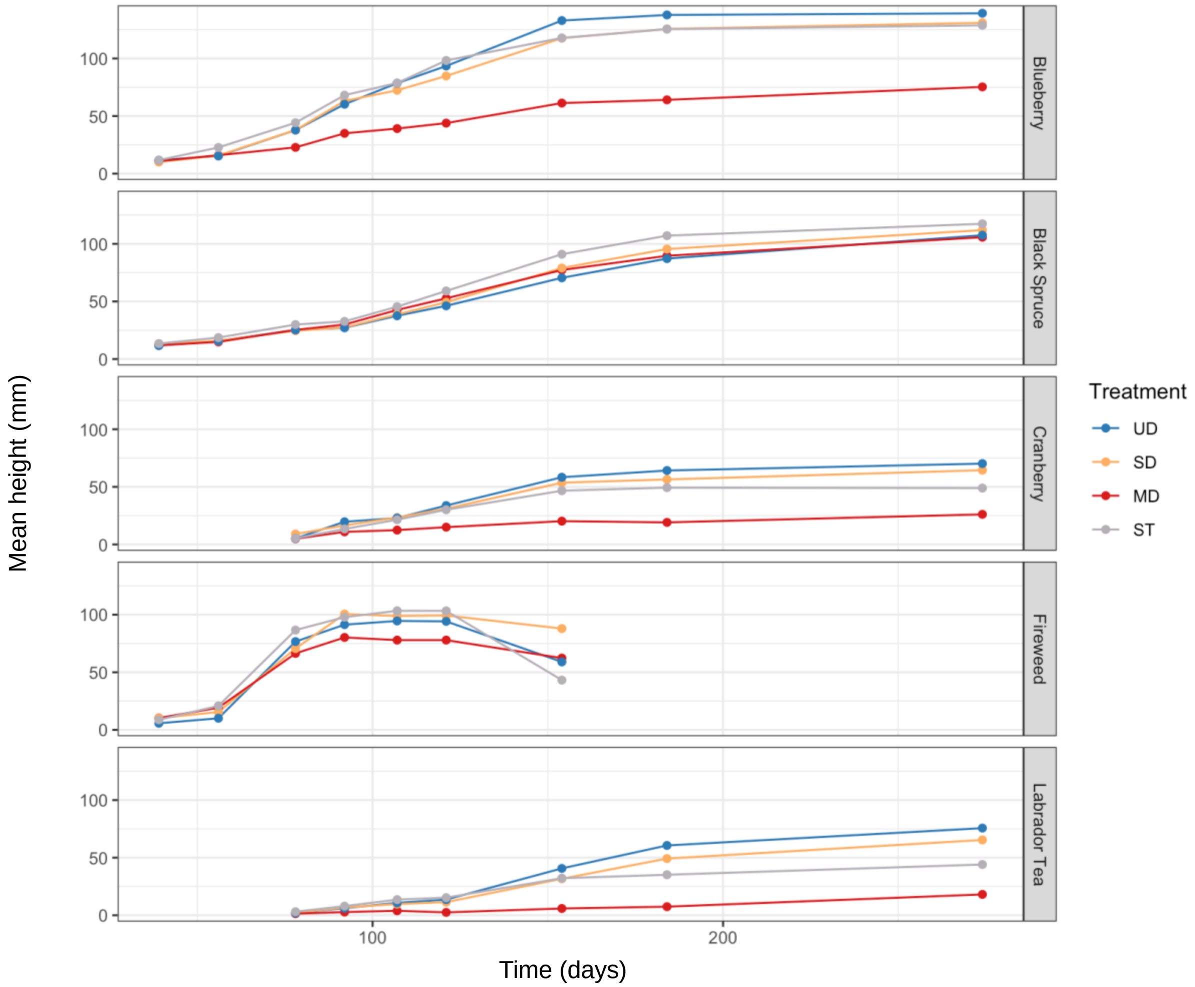

Supplementary Figure 2. Average height over time for each plant type. Fireweed growth ends on day 121 when the plants were harvested for above ground biomass
