# Supplementary material for: Soil Disturbance Affects Plant Productivity via Soil Microbial Community Shifts
Source: Front Microbiol. 2021 Feb 1;12:619711. doi: 10.3389/fmicb.2021.619711 (PMC7882522; doi:10.3389/fmicb.2021.619711)
Supplement: Supplementary file 12 [file Data_Sheet_3.pdf]

Supplementary File

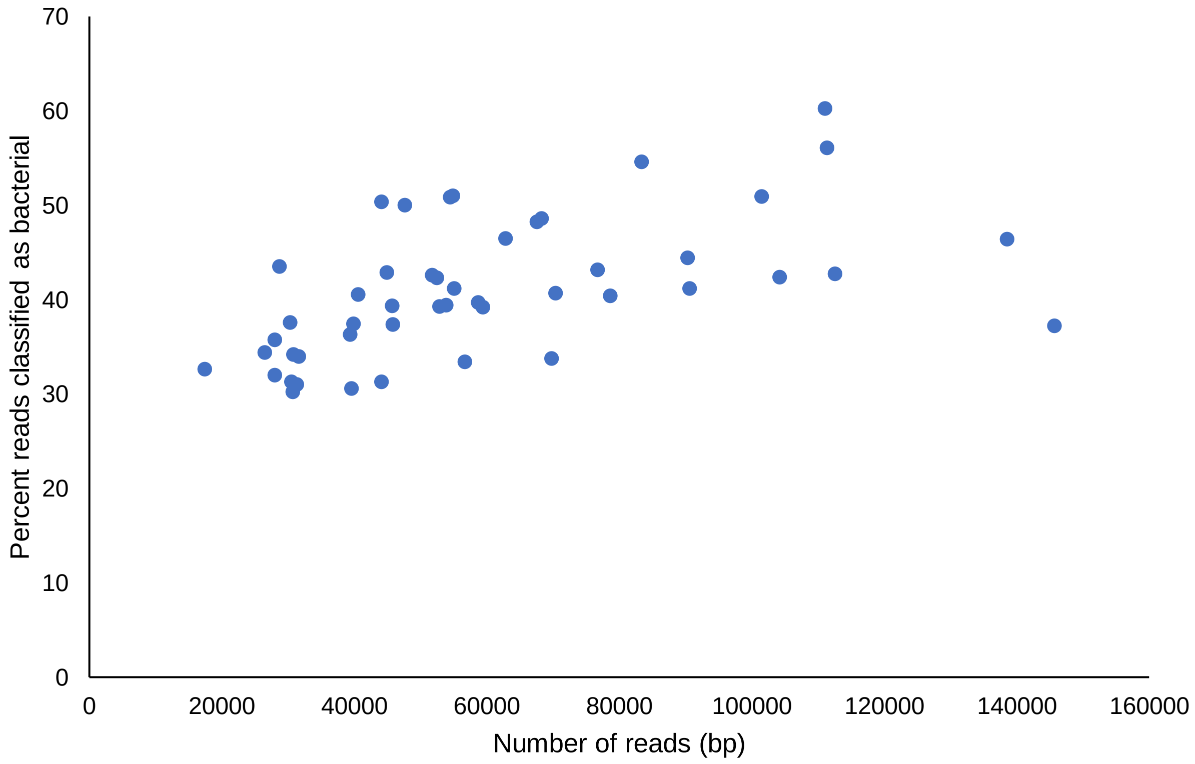

Supplementary Figure 3. Percentage of metagenomic reads classified as bacterial and the number of reads classified via Kraken2 (n = 48)
